# Exploration of Finnish adults’ successful weight management over the life course: a qualitative study

**DOI:** 10.1186/s12889-019-8128-8

**Published:** 2020-01-06

**Authors:** Anu Joki, Johanna Mäkelä, Hanna Konttinen, Mikael Fogelholm

**Affiliations:** 1 Department of Food and Nutrition, University of Helsinki, P.O. Box 66, 00014 Helsinki, Finland; 2 Department of Education, University of Helsinki, P.O. Box 8, 00140 Helsinki, Finland; 3 Sociology, University of Helsinki, P.O. Box 18, 00014 Helsinki, Finland

**Keywords:** Successful weight management, Normal weight, The life course perspective, Qualitative research, Self-efficacy, Habits

## Abstract

**Background:**

Despite the current obesogenic environment creating challenges weight management, some people succeed in maintaining a normal weight. This study explored lifelong weight management from the life course perspective. We aimed to gain an insight into the issues related to the pathways of individuals of normal weight from childhood to adulthood, and how their experiences and social connections influence their weight management.

**Methods:**

We approached the research topic using qualitative methods. Two age groups (30–45; 55–70 years, men and women), forming a total of 39 individuals, participated in theme interviews. Thematic analysis resulted in two main categories, namely (1) adoption of lifestyle and (2) maintenance of lifestyle.

**Results:**

Childhood family played a central role in the formation of lifestyle: food-upbringing created the basis for the interviewees’ current diet, and their lives had always been characterized by an active lifestyle. High perceived self-efficacy was vital in weight management. The interviewees were confident about their routines and trusted their abilities to recognize and handle situations that threatened their lifestyles. They possessed skills for adjusting their lifestyle to altered environments, and showed a high level of coping self-efficacy. The interviewees also highlighted the importance of habits for weight management. They had improved their adopted lifestyle through constant learning. New routines had become more internalized through active repetition, finally turning into habitual practices, which simplified weight management.

**Conclusions:**

Based on our interviews, we conclude that childhood was important in the development of the health-promoting lifestyle of our interviewees. However, weight management was described as a journey over the life course, and success also encouraged skills of identifying risks and adjusting actions to cope with challenging situations.

## Background

Obesity, with its co-morbidities, threatens public health in both developed and developing countries [1]. Weight control attempts (weight loss, maintenance, or both) have become more prevalent in the current obesogenic environment, and results from a recent systematic review and meta-analysis (covering 72 studies) showed that 42% of adults worldwide try to lose weight and 23% try to maintain their weight annually [2]. A growing body of literature has demonstrated that weight maintenance requires continuous work. Individuals who have successfully maintained weight loss have regular meal patterns and follow a healthy diet, moderation describes both their energy intake and portion sizes, and they are highly physically active [3, 4]. They are constantly conscious of their eating routines and activity levels. Flexible eating control with moderate eating restrictions also plays a major role in success [5]. Women of normal weight (body mass index, BMI, between 18.5 and 24.9) described weight management as “work” that they struggle with [6].

Numerous population studies have explored the factors (determinants) that contribute to changes in weight [7, 8]. Several studies have also examined weight management after weight loss, using an intervention design [9]. Nevertheless, these studies have typically concentrated on predefined hypotheses regarding the associations of certain behavioral and psychosocial factors with weight change on a group level. It is uncertain whether this kind of approach can determine more complicated, individual issues. To obtain a deeper insight into weight management, we believe that population-based quantitative data should be enriched by individual-based, hypothesis-free approaches.

Our previous paper introduced individuals’ views and thoughts on lifelong weight maintenance success at the present time-point [10]. We defined lifelong weight maintainers as those who had sustained normal weight for their entire adult lives. These maintainers self-regulated their behavior in a permissive, flexible and conscious way. Although their success required work, they did not perceive it as challenging; instead they saw it as a way of taking care of themselves. The informants felt that lifelong weight management was not restrictive; it increased the quality of life.

As, our first analysis did not clarify when and how successful means of weight control were formed [10], we wanted to find which factors played the most central role in developing the routines and habits that guaranteed success. Interestingly, our participants repeatedly mentioned childhood in various contexts, and this idea became our starting point for the present analysis and study. Consequently, it expands our knowledge and examines new aspects of successful weight management.

We also wanted to consider the meaning of self-efficacy over the life course, since many previous studies have connected self-efficacy to successful weight management [3, 11]. Our first analysis implied that self-efficacy might play a role in lifelong weight management, and the fact that self-efficacy and the related concepts are important elements of several health behavior models (e.g. Health Belief Model, the Theory of Planned Behavior, and the Health Action Process Approach) further reflects the central role that self-efficacy plays in human behavior and functioning [12]. Thus, based on these reflections, we decided to add the concept of self-efficacy to our analysis. We aimed to identify common features in our participants’ pathways: how they had adopted their lifestyles and how they perceived the influence of earlier experiences on their current behavior.

### Theoretical background

#### Life course

The life course perspective follows individuals’ lives over time; it sees life as a trajectory or a pathway [13]. The life course perspective is a set of different theoretical approaches and explanation models, and it contains five principles: 1. Human development and aging as lifelong processes, 2. Human agency, 3. Timing, 4. Linked lives, and 5. Historical time and place [13]. The first principle refers to the relevancy of earlier experiences for understanding individuals and their choices and behaviors in the later years of their lives. Due to the nature of life course as a cumulative process, studying life in its entirety, rather than as single events, is recommended. The second element, human agency, indicates situations in which people select certain roles and positions in order to achieve their goals [13]. However, social and historical environments often determine the frames in which individuals can act. The principle of “timing” considers normative expectations of when a certain event or transition occurs in a person’s life. The “linked lives” principle in turn is related to the idea that all people touch the lives of others. Family relationships, especially the connections between parents and children, are also relevant. The last principle, historical time and place, refers to the fact that everyone is part of a certain birth cohort. Social variations in the experiences of different cohorts may affect the health of different cohort members.

This work investigated maintainers’ pathways from childhood to adulthood in the context of the current obesogenic environment, and how social interactions and weight maintainers’ personal development enabled their success. We did not focus on the “timing” principle due to a lack of exact information on the nature and timing of the transitions that occurred in the participants’ lives. Understanding the experienced transitions was sufficient for us to meet our aims.

#### Self-efficacy

Human agency is one of the main principles in the life course perspective. It also connects the life course view and self-efficacy. Perceived self-efficacy, which describes people’s beliefs regarding their capabilities of succeeding despite various challenges, influences health behavior at every phase of the life course [14]. The mediating mechanism may be self-efficacy, which modifies individuals’ capabilities of organizing learning skills and motivation for achieving their goals, all of which are elements for which human agency is responsible. As Bandura stated [14], individuals with high perceived self-efficacy pursue challenging goals, pursue them with strong commitment, and finally achieve the goals they set. When compared to people with low self-efficacy, individuals with high self-efficacy are generally healthier, and manage to simultaneously cope with a number of challenging situations such as the requirements of family, work or several school assignments, and are more successful in achieving various educational, career- and leisure time-related aims [14–16].

Four main factors are involved in the development of perceived self-efficacy [14]: mastery experiences, social models, social persuasion and individuals’ emotional and physical states. The successes experienced through effortful tasks create robust belief in individuals’ personal efficacy. Self-efficacy also increases via encouragement from other people or by recognizing success among people similar to oneself. Furthermore, mood affects individuals’ beliefs regarding their efficacy. However, it must be pointed out that self-efficacy is behavior- and context-specific, and might be high in some issues but, low in others [14]. For example, an individual might have high self-efficacy in maintaining a physically active lifestyle but simultaneously low self-efficacy in maintaining a healthy diet.

Moreover, self-efficacy is not a constant feature; it varies over the life course. Family and peers shape initial efficacy experiences, which school and learning further extend [14]. Life transitions present individuals with different challenges and force them to make adaptations to their established routines in order to maintain their functioning. The higher the sense of coping self-efficacy, which describes an individual’s perceived capabilities of coping in challenging situations [14], the better the surveillance of those challenging situations. In summary, the development of self-efficacy is an ongoing process.

This study aims to explore the pathways of individuals of normal weight from childhood to adulthood and how their experiences and social connections have influenced weight management.

## Methods

This study was based on 39 semi-structured theme interviews conducted in Finland. We determined successful weight management as the maintenance of normal weight (BMI, between 18.5 and 24.9) during one’s lifetime (early adulthood to present age). To find appropriate participants, we used a purposeful sampling technique for identifying and selecting individuals who were particularly knowledgeable about the phenomenon of interest [17]. We began by recruiting participants from two large organizations representing typical Finnish public employers: the Public Works Department of the City of Helsinki and the parish union of Helsinki, as the principal investigator (AJ) had contacts at these workplaces. First, we mailed the invitation letter, which contained information on the research and criteria for participation, to two employees at these workplaces, who then forwarded it to the potential participants. Next, the principal investigator (AJ) emailed each eligible and interested candidate and gave them detailed information on the research. The study therefore utilized the snowball sampling method, which is useful when informants are members of certain subgroups [17]. We asked the participants to suggest other potential future participants from among their acquaintances. In addition, the principal investigator (AJ) promoted the study in her social networks to recruit additional participants. However, she did not interview anyone she knew personally well; only acquaintances. It is also noteworthy that although we began the recruitment process in public sector organizations, the sampling method used enabled us to reach informants from various employers, even some individuals working in the private sector. To specify, only 10 of the 39 informants worked in either the Public Works Department or in the parish union of Helsinki.

The present study examined men and women from two different age groups (age 30–45 and 55–70 years) who had always been of normal weight. These age groups represented two birth cohorts who had experienced a different kind of childhood: the older group consisted of “baby boomers” who were children in the period after the Second World War, and the younger group members belonged to the baby boomers’ children’s generation. During the war, food was rationed in Finland. The parents of the older age group had experienced national shortages. However, after the war, the standard of living rose, and the childhood food environment of the older age group changed from one of shortages to one of abundance. In contrast, the childhood environment of the younger birth cohort was characterized by emerging nutritional recommendations that guided individuals to make their eating behavior healthier, as obesity and other health problems had emerged [18]. Since 1970, the selection of available foods had also become more diverse. To conclude, the childhood food environment of these two age groups differed from each other.

Table 1 presents the characteristics of the participants. The data are based on self-reporting. Their average BMI was 22.6 (range 20.1–24.9). Almost all were moderately physically active: They reported exercising several times per week. Most of them had studied for at least 13 years (including school years), but education levels varied somewhat. It should be noted that in Finland, the average increase in education levels since the Second World War has been rapid [19]. The educational level of the participants’ parents, particularly that of the older age group’s parents, was lower than the educational level of the participants themselves. Their occupations and family relationships also varied, as did their physical abilities. Thus, this was a heterogeneous group, which shared weight management success. It contained two parent-child pairs and two spouse-pairs.

**Table 1:** Characteristics of study participants by age group

	30–45 y	55–70 y
Gender:		
Men	9	10
Women	10	10
Education:		
0–9 y	0	3
10–12 y	3	5
13 y or more	16	12
Have a child or children:		
Yes	16	19
No	3	1
Smoking/ use of snuff:		
Yes	3	1
No	16	19
BMI	22.3	22.9
Leisure-time moderate-to-vigorous physical activity:		
Daily	0	5
4–6 times/week	3	3
2–3 times/week	15	9
Once a week	1	1
Less frequently	0	2

We used semi-structured theme interviews as a data gathering method. These flexible interviews adapt the interview structure to suit each interviewee [20]. To create the interview themes, we used the results from studies of weight loss and weight management [3, 21]. Three pilot interviews were conducted to test the interview structure. Our previous text contains detailed information on the pilot interviews and actual theme interviews conducted [10]. The interview discussion topics covered the participants’ relationship with food and eating as well as their perceptions of the connections between weight management and health behavior. The interviewees also defined weight and eating management and reflected on the factors associated with their weight.

The principal investigator (AJ) interviewed all 39 interviewees. The interviews, based on an interview guide (Additional file 1), lasted from 45 min to two hours, and were conducted in various settings chosen by the informants: the participants’ homes [20], workplaces [16] or the University of Helsinki [3]. A digital voice recorder was used to store all the interviews, which were subsequently transcribed verbatim, and totaled 463 pages. All interviews, transcriptions, and the analysis were conducted in Finnish. The quotations used in the current article were translated into English by a professional native British language editor.

As the field of this research topic is still fairly unknown, we decided to use thematic analysis to yield results. This analysis method is suitable for great amounts of textual data and fields with limited research knowledge [22]. Moreover, thematic analysis enables a deeper understanding of the studied phenomenon, as the method not only describes the phenomenon, but seeks an interpretive level with which to reach the underlying meaning of the text [23]. It also provides a flexible and practical method for expanding knowledge regarding the human experience of health. For systematic data analysis, we used Atlas.ti software as assistance [24]. First, the transcribed data were entered into the program. After familiarization with the text, the analyst (AJ) coded the text quotations that described the relevant information concerning the research questions [22]. During the coding process, the research data were conceptualized and classified into meaningful and relevant categories for data analysis and interpretation.

In this theory- and data-based analysis, the initial coding process followed the categorizations of the life course perspective by forming three codes (childhood, adulthood, transitions), the text quotations of which we classified. During the coding process, we realized that it was necessary to create sub-codes such as “childhood and eating” or “transitions and linked lives” to facilitate the process of analysis. In order to secure the reliability of the analysis, we used a coding list that included explanations of the codes and sub-codes [23]. We also paid attention to the other principles of the life course perspective during the coding process (human development as a lifelong process, personal development, social interactions, historical time). The researchers discussed and evaluated the adequacy of the coding, and based on the data, decided to add new codes (learning, adjusting) (Additional file 2).

Then, in the interpretation phase (meaning-making process), after careful revision and several rigorous coding rounds, the relations and similarities among the codes formed two main categories: Adoption of lifestyle and maintenance of lifestyle. The former included the “childhood” and “learning” codes, whereas the “transition”, “adulthood” and “adjusting” codes belonged to the latter. For example, text referring to the development of the participants’ food habits or active lifestyle were assigned to the “Adoption of lifestyle” category and text referring to adjusting habitual routines to new life situations were assigned to the “Maintenance of lifestyle” category.

## Results

The following paragraphs describe the main findings of the present work. First we introduce the issues related to adoption of lifestyle, and then continue with factors connected to the maintenance of lifestyle. However, it should be noted that the themes overlap, and adjusting or learning cannot be found at any particular stage of life, as they may be present in various stages of the life course. Within these main categories, we focused on the pathways that successful weight maintainers had experienced, and elicited their views and beliefs regarding childhood, transitions and adulthood.

### Childhood

Both age groups highlighted the central meaning of childhood in creating lifestyle, despite the different childhood landscapes that they had lived. The interviewees perceived that they had adopted their weight gain protection values and attitudes from their parents: “Of course, your childhood family have influenced it … it’s the lifestyle that you’ve grown up in and what you’ve learned … also, my mom and dad have always been active and eaten healthy foods and been conscious of their weight control routines” (I22W34).^1^ Some minor differences occurred between the age groups: older participants reported that during their childhood, their parents, who had experienced national shortages, emphasized the importance of food and learned not to leave food on their plates. The younger participants did not find it so difficult/uncomfortable to leave food uneaten if they were full.

#### Food-upbringing

Childhood family and its routines formed the basis of food patterns. This effect, which we defined as food-upbringing, played a significant role in the interviewees’ current food habits. They reported eating the same traditional Finnish foods that were familiar from childhood. More specifically, in everyday life, they cooked similar foods to those they were served in their childhood homes. Their favorite foods also contained typical home-cooked meals such as “macaroni bake” or “meatballs and mashed potatoes”.

The interviewees also felt that their taste preferences had developed in childhood. As one 31-year-old woman explained: “Starting from childhood … manners and habits and preferences, also taste preferences, originate from childhood” (I2W31). Vegetables, especially root vegetables, had been regularly eaten by the interviewees and the consumption of sweets had been infrequent in their childhood. Consequently, vegetables seemed to play the main role in the interviewees’ current diet, and their consumption of treats was moderate. Their meal frequencies corresponded to their childhood meal rhythms. Regular meal patterns and proper meals characterized their eating habits in both childhood and adulthood. As one 31-year-old man described: “Upbringing is the main element that influences my food habits. I can’t think of any other factors, I’ve noticed that I eat the same foods as I did in childhood. I also think about what my mom and dad taught me about food …” (I8M34).

Even the relationship with food seemed to have been born in childhood. The interviewees appreciated food simply as an energy source, but they also highlighted its meaning in celebrations and social interactions. Their relationship with food seemed to be uncomplicated: food neither caused anxiety nor guided their lives; it was important in a positive way. It was uncommon to combine food and feelings – food was not a reward but a tool for living. They emphasized their mothers’ role in creating a healthy food relationship. They had regular, proper meals in childhood. Food was homemade, tasty and not “snobby”. Several stories pictured a mother slicing apples, carrots and other fruit for breakfast or making soup for a family meal. Food memories also described special moments: “camping in the woods and cooking on a campfire with dad” (I8M34) or “the harvest festival where the entire village gathered to dig potatoes and the tables were weighed down with all the different kinds of foods that mom had cooked” (I34W62).

#### Variation in socioeconomic background

The interviews provided new insights into the socioeconomic background of the interviewees. The interviewees highlighted how food was appreciated in their childhood families and was prioritized when money was short. As one 64-year-old man explained: “When I was young, we didn’t have much money. I lived with my mom; my dad left when I was a baby. My mom had to work hard to get me food. And I was only 15 years old when she died.” (I17M64). Another participant explained: “As a child, I was very skinny. I had a second cousin who was the same age and she was plump. Her family had money to buy sweet things … she gained weight and I always asked my mom, how I could also gain some weight … we never had anything sweet, only homemade food but if I think about it now, it was good, healthy food” (I39W60).

Several interviewees, particularly the older participants, mentioned that when they were children their family could not afford to buy expensive vegetables and treats, but that the food they ate was still healthy: “Now I eat various vegetables. However, if I think about my life as a whole, supply was more limited when I was a child. Vegetables were from our home garden, such as carrots, beetroots, peas, cabbages … we couldn’t afford to buy vegetables.” (I15W60). However, the younger interviewees also gave examples of how their families carefully thought about what to spend money on, and food was something they wanted to invest in.

#### The adoption of lifestyle

The interviewees believed that genes influenced their weight management, but they also proposed that (in addition to genes), their lifestyle was inherited by learning. They reflected on their relatives’ weight and lifestyles and concluded that success was not only determined by genes, but that lifestyle was a relevant factor. “Genes are what they are, and it’s hard to comment on them. However, I feel that in my case, the behavior model from childhood is more significant than genes … my parents have always been physically active and controlled their eating …” (I37M62).

The interviewees also revealed that not all the eating habits from their childhoods promoted successful weight management. Nevertheless, they had recognized these poorer habits and created solutions. One 32-year-old woman specified: “We ate a lot, probably too much, in my childhood family … some of my relatives are overweight, my mom and one sister for example, and it makes me restrict my eating now …” (I31W32). A 37-year-old man explained, “Especially at Christmas time, I find myself taking candies from the kitchen cupboard … I remember from childhood that we had a lot of candies and pastries to eat for several days … I want to stop this behavior model by not keeping treats in the cupboard” (I6M37). Therefore, when food-upbringing did not support the interviewees’ goal of a healthy lifestyle, they were able to ignore their adopted habits and adjust their behavior to reach their goals.

#### Physical activity and an active lifestyle

The interviewees had grown up with active lifestyles. They described having always been interested in exercise and that it had been a crucial part of their lives since childhood. The age groups mentioned different ways of implementing physical activity in everyday life, but the importance of physical activity was similar in both groups. As one 30-year-old man summarized, “I’ve had an active lifestyle since childhood … I didn’t spend time on the computer or play video games; I exercised frequently and ate regularly and most likely fairly well” (I32M30). A 65-year-old woman explained: “I cycle, swim and go for walks … I’m not a gym bunny, but I need some physical activity … just for fresh air and to compensate for days spent inside at work” (I16W65).

The interviewees emphasized “an active lifestyle” (constant motion and bustling) as being more important than having a certain sports hobby. Of course, an enjoyable hobby was beneficial for weight management, but success did not require any specific one. Exercise also offered the interviewees many other advantages (mental well-being, stress relief, endorphins), and weight management was only a side-product. A 39-year-old woman clarified: “Yes, I think exercising is important, but not only for weight management. In general, it helps well-being, and helps you keep working, helps you keep in shape. It enhances body awareness, and I truly believe it’s the key to everything” (I7W39).

The maintainers’ social ties shared this healthy lifestyle. In childhood, the interviewees’ parents, especially mothers, who were praised in several interviews, played a crucial role in generating eating and exercise practices. As one 62-year-old man explained: “My mom was a skillful cook and she prepared healthy, diverse food even though we didn’t have much money when I was young.” (I37M62). Because of this early social context, the interviewees were already capable of exercising and making healthy food choices as children. Their social networks had been active since childhood, and many of them were also of normal weight. As one 37-year-old man stated, “My social network doesn’t really affect my weight management … sure, my friends are active, they exercise and are in good shape … so, it may have an influence, but I can’t really say it’s down to that because it’s the environment I grew up in” (I23M37).

### Learning as a continuous process

The interviewees had learned and adopted new routines continuously throughout their life course. Active habituation played a major role in their learning processes. Conscious choices and decisions turned into internalized behaviors by repetition. Habits that had been implemented since childhood had become routines during the life course. One 67-year-old man told us, “I think that the main reason for being of normal weight is the lifestyle that I learned from home. When I was young, there weren’t many food choices, you had to eat what was on offer, and it was mainly healthy food. Hamburgers or pizzas weren’t available, and I wasn’t used to those kinds of foods. So, it’s uncommon for me to eat junk food. I rather eat healthy home-cooked food, it’s the lifestyle that I’m used to, and I don’t have to think about it!” (I29M67). A 56-year-old woman also highlighted the importance of habituation in the formation of routines, “I just eat the same way I’ve always eaten … it’d be hard for me to change my routines and start to overeat or stay on the sofa instead of going out for a walk” (I1W56).

Although habits were adopted in childhood, learning continued into adulthood. Conscious decisions to behave in a certain way in order to achieve a goal, such as healthy living, had changed over time into routines that no longer needed to be considered. One 61-year-old woman explained, “I love salads, I could eat them all the time … but if I think of my favorite foods now, I admit I might have programmed myself to this kind of diet at the beginning … however, I like my diet now” (I36W61). Another example of active habituation in the adoption of a lifestyle was: “I hardly think about weight management anymore … I don’t feel at all that I should restrict my eating or my life or anything, because those patterns are now so internalized … but it didn’t happen by chance … in the beginning, I decided to behave in a way that promotes staying at a normal weight … and now the routines just happen” (I12W64).

Habitual practices did not require continuous work. As some routines were currently automatic to the interviewees, it was unnecessary to think of food choices or meal sizes because they had already earlier learned how much they needed to eat. Possibly for this reason, participants perceived weight management as rather effortless. Several interviewees detailed how they did not work at weight management but maintained a lifestyle that obviously enabled success. A 58-year-old man explained: “I don’t need to think about weight management; it just happens routinely due to my lifestyle. And that’s the main point! At the end of the day, I work out quite little nowadays, but I still need some sport. I have a compelling need for outdoor sport; if nothing else, I have to go for a walk. But I don’t need to think about these things!” (I9M58).

### Adjustments during life course transitions

The participants encountered events and situations in their life courses that threatened their characteristic lifestyle. Typical transitions that they mentioned were marriage or divorce, pregnancies and parenthood, starting a new job, and spending a longer time abroad. During these transitions, the interviewees worked actively to maintain their weight and successfully balanced energy intake and consumption. Instead of carrying out given instructions they modified their behavior on the basis of their earlier experiences and reflections.

The interviewees mentioned several examples of times when they had needed to make such adaptations. One 39-year-old woman described her experience, “When I began this desk job, I noticed (from my body) that I didn’t need to eat as much as I used to. My previous work had been physically harder, and at that point, I had to consciously ‘wake myself up’ and realize I would survive with less food … meals didn’t need to be huge … in conclusion, I’ve always found the right balance for my needs in this kind of situation.” (I7W39).

The interviewees succeeded in tackling threatening transitions and returning to their customary lifestyle. They recognized and intentionally reacted to situations in which they approached their adopted upper weight limit. They wanted to maintain their ideal weight and were ready to work to achieve this goal. They actively cut off the chain of weight gain: “I was an exchange student in America when I was young. During that year, I gained weight … because of the American lifestyle … and when I returned, I continued my Finnish lifestyle (walking and cycling, eating home-cooked food) with some extra means of weight control like avoiding treats, and I lost the extra kilos” (I35W57). Another quotation containing several examples of this kind of situation was: “I’d always been slim … but almost 15 years ago, a strange thing happened; I was almost eight kilos heavier than now but still of normal weight … I’d just gained some weight, the kids were small, and I was studying, and I was exercising very little … then I realized that if I continued in the same way, I would have to buy the next clothing size and this was the last thing I wanted … so I increased my exercise and was more conscious of my eating … and I lost the extra kilos … it was quite easy …” (I14W56). Achieving and maintaining set goals were characteristic of the individuals who had successfully maintained their weight. Naturally, there were several ways to maintain their ideal weight and the interviewees had made adaptations that suited them best.

In contrast to the above examples, some transitions supported weight management. Parenthood was a central milestone at which the interviewees revised their routines and habits. As a role model, they offered healthy foods, ate regularly, organized family dinners and were physically active. One 37-year-old man explained: “When I was a young man, I ate differently, a lot of unhealthy processed foods, hamburgers and hot-dogs … when I had children, I changed my diet … now it resembles my childhood diet, which included plenty of healthy things” (I23M37). They also highlighted the importance of initially learning healthy food and exercise patterns.

### Self-regulation, routines and social environment as supportive factors

The interviewees described weight management as an issue they could control to keep their weight stable. They believed they were responsible for their decisions and self-regulated their behavior, despite unhealthy temptations in their environments. As one 34-year-old woman told us, “Well, my husband quite often eats chips on weekends, I typically take two handfuls, and then I quit … I make my decisions … of course, the environment has some influence on people’s patterns, but I think, ultimately, you yourself are in charge of your eating” (I22W34). They also pointed out that everyone faces difficulties in life but that the attitudes and reactions in those situations are more significant in determining results such as successful weight management. They did not allow problems to disturb their weight maintenance.

The interviewees showed confidence in their lifestyles and routines. External instructions, fad diets (a diet promising rapid weight-loss) or even family or close friends did not regulate their habits: “… I am so routinized, and we have a basic set that we always eat …” (I2W31), “… no effect, my thoughts related to food are so stable/constant …” (I3W41). They wondered why people are so uncertain of their own routines and let different kinds of fad diets (which maintainers saw as ridiculous, unnecessary and short-lived) change their food habits. However, despite the “certainty” characterizing the interviewees’ lifestyles, they were not entirely rigid in their routines. They carefully considered if a new habit would be meaningful to them and subsequently, sometimes changed their behavior. A 61-year-old man explained: “I didn’t like fish in the past but when I heard how healthy and good fish oils were I just started to eat fish … I also drink sea buckthorn juice daily and carrot juice … they’re not my favorites, but good for health … and after all, I’m used to them now” (I33M61).

The social environment of the interviewees was mainly favorable for their weight management. Due to their unproblematic relationship with weight management, they felt no need for support. However, they recognized that their spouses had similar lifestyles and identical values. In a way, they received “passive support” when their family ate healthy foods and exercised together. Their relatives respected their lifestyle, and in most cases also shared it. As one 37-year-old man described, “I find it the main issue … the whole family matters … your lifestyle may be part of the whole family’s lifestyle … I can imagine that if my wife didn’t care about her eating, I’d easily go along with her …” (I6M37). However, in conflicting situations, the interviewees retained their views.

Adjustments also occurred in everyday life, not only during special transitions. The immediate reaction to weight gain was seen as crucial to success. The participants underlined that it was essential to respond to even a small weight gain – this was the key to adjusting one’s lifestyle to the new environment. Customary food habits were modified: “My metabolism isn’t as effective as it used to be, and so I’ve changed my eating habits … I eat more salads, and I’ve replaced potatoes and rice with cooked vegetables...sedentary work doesn’t help, so I’ve made some adjustments to prevent weight gain” (I28W31). The interviewees made small adjustments to continue balancing their long-term weight management.

## Discussion

Our interviewees’ stories show us that weight management is a continuous process; a journey through the life course. Childhood played a key role in the adoption of a lifestyle that promoted weight management. Regular eating, a vegetable-rich diet, and an active lifestyle characterized maintainers’ practices over their life course. High coping self-efficacy was necessary for success. We concluded that the interviewees had an ability to adjust their lifestyles to an altered environment. According to our interpretation, behind their success were weight management habits that were rooted in everyday routines throughout the life course.

### Food-upbringing

Food-upbringing, by definition, describes the role of parents in shaping their children’s early experiences of food and eating. It formed the basis of the interviewees’ current diet. As the life course perspective suggests [13], family relations during childhood modified their choices and behavior. The lifelong weight maintainers repeatedly emphasized the vital role of childhood in creating their lifestyles. The interviewees’ parents, as role models, shared a lifestyle that supported weight management, and as they were responsible for what foods were available at home, they offered healthy foods such as vegetables to their children. According to our interviewees, mothers played a significant role in generating the successful maintainers’ healthy relationship to food. It is extremely interesting that two age groups that had experienced different childhood landscapes both highlighted the “power of their mother” and the importance of childhood family in the development of their lifestyle. However, it should be noted that the role of the mother as the person with the principal responsibility for food and cooking was more significant 30 years ago than it is in today’s society in which fathers also play an essential role in food-related matters [25]. Thus, despite the fact that the current study emphasized the role of the mother, fathers’ significance in the creation of lifestyle should not be underestimated.

Socioeconomic background is closely related to an individual’s life opportunities, health values, perceptions and practices [26]. In the Nordic Countries, educational history has shown to be related to differences in social backgrounds, which further indicate differences in eating habits and obesity levels [27]. Accordingly, it could be assumed that the social status of the participants and a good, privileged childhood may explain the findings of this study. However, based on our data, this may not be the case. Even though most of the interviewees had a high social and educational status at the time the study was conducted, many did not have privileged childhoods or upper-middle class families according to their stories. The educational status of the parents of the interviewees was lower than that of the interviewees themselves. Furthermore, the family background of the participants varied from “traditional” families to single parent families*.* Therefore, it is interesting that successful weight management also seems possible for individuals who have grown up in a less advantaged environment.

The home food environment and parental behaviors, which the present study conceptualized for food-upbringing, have an impact on children’s behavior and adult weight [6, 28–33]. Previous cross-sectional studies have shown that parents can influence the nutritional quality of their children’s diets by encouraging and modeling healthy eating, forming “rules” for foods that are allowed and limited in the home and keeping nourishing foods easily available [29, 34]. Children’s health practices seem to follow their mother’s health behaviors [31], and eating behaviors are shaped by both parents [30]. Parents also affect the development of their child’s food preferences and energy intake [32].

The lifestyle learned in childhood seems to remain over the life course. We recognized that our interviewees’ lifestyles had always been active, and that their diets resembled those consumed in childhood. Previous studies have acknowledged this same phenomenon. The dietary and physical activity routines [35] and health beliefs and behavior [36] learned from the family tend to persist throughout adulthood [35, 36]. Women of normal weight also consider family background important [6]. According to a tracking study of physical activity, an active lifestyle adopted early in childhood remains stable from youth to adult age [37]. In addition, parents’ high levels of physical activity are positively associated with their children’s activity levels [38]. According to Kaseva et al. [38], the beneficial effect on offspring’s lifestyle continues to at least middle age. The present study highlights the centrality of continuous activity in successful weight management.

Finally, previous studies have presented the main strategies for successful weight-loss maintenance [3, 5, 39, 40]. Accordingly, multiple weight control practices (restricting dietary intake, monitoring weight, eating low-fat foods, exercising intensively) were significant, and weight-loss maintainers needed to observe their weight management carefully. Moreover, some described weight management as still being a struggle, even years after losing weight [40]. The present study found that instead of strict rules and laborious control, success required habits that were adopted throughout the life course. We interpreted that weight management was the consequence of an individual lifestyle rather than of a definitive period of hard work.

### Self-efficacy

According to our interpretation, the interviewees’ beliefs and behavior in terms of issues related to weight management illustrated high self-efficacy. Successful weight maintainers believed they were responsible for their choices and routines and were committed to their lifestyle and ready to work at it. Moreover, their confidence in their lifestyle and the internalized routines they performed was strong, as was their trust in being able to cope with challenging life events. These findings fit the theory of a healthy lifestyle, which states that the development and maintenance of a healthy lifestyle is a complex process, influenced by multiple factors [41]. Features such as self-efficacy, feeling in control of one’s life and a having variety of possible choices play a role in the maintenance of one’s lifestyle [26, 41, 42]. Lifelong weight maintainers had these features and believed in their capacity to maintain their healthy lifestyles.

The interviewees’ high self-efficacy in issues related to weight management had developed during their journey along with the successes they achieved. To ensure the maintenance of their set lifestyle, they continuously adopted new approaches to learning, thus even improving their lifestyle. They had learned to trust themselves in situations in which they needed to check a course of action and possibly adjust their lifestyle to the changing environment. They were also good at identifying weight gain risks. As these skills are related to the coping self-efficacy concept, we interpret that self-efficacy played a vital role in the maintenance of our interviewees’ lifestyles.

Self-efficacy is also essential in life course transitions, which might be “stumbling blocks” for weight management. Combining family and working career is a common issue, and parenthood is identified as a period of great modifications [14]. Limited resources threaten weight control, and without tenacious self-efficacy, which enables coping with challenging adversities, weight gain is predictable. A qualitative study of mothers of normal weight mentioned childcare as a barrier or at least a challenge to successful weight management [6]. However, according to our interviewees, being a role model for children supported also their own weight management. They wished to change their routines, such as regular eating, preference for healthy foods and a physically active lifestyle, for their children, which had an impact on the maintenance of their current lifestyles. Our interviewees adjusted their behavior in even healthier directions in these circumstances. Thus, we suggest that with high coping self-efficacy, lifelong weight maintainers overcame challenging transitions and succeeded in remaining at their ideal weight.

### Habits

An exciting finding of the present study was the major role that habits play in successful weight management. Phenomenological studies define the term “habits” as present bodily actions that past experiences have shaped into situationally adequate and adaptive actions [43]. Earlier interactions and learning processes produce these habits, and these should be distinguished from rigidly automatic processes such as reflexes. Interestingly, our results fit this phenomenological definition. The interviewees did not actively think about food choices and meal sizes or whether to go out for a walk, because these were routines that they were used to following. However, in the beginning, these habits needed more conscious actions. According to our interviewees, as learned and internalized habits occur routinely, this did not require constant work or assisted weight management.

Health behavior is known to be the sum of conscious and automatic processes [44]. Our study came to the same conclusion, as we recognized the importance of both these factors in successful lifelong weight management, self-efficacy illustrating conscious processes, and habits describing automatic processes. The interviewees saw weight management as possible, and their established routines stabilized any needs to control their behaviors. This may also explain why the interviewees did not perceive weight management as difficult. In contrast to our results, one study has found that familiar customs do not facilitate the weight control of women of normal weight: these women experienced it as laborious [6]. They also mentioned that weight control demanded regimentation in exercise and discipline in food choices. The attitudes toward weight management differed between our interviewees and women of normal weight. Lifelong weight maintainers identified weight management as favorable and understood its role in promoting health and life quality.

One of the current interests in health psychology research concerns habits and their significance for health promotion and weight management [45, 46]. The present study interprets that habits play a key role in the success of weight management and facilitate it because they have been implemented since childhood. Researchers who are developing behavior change interventions are intensively exploring the formation of habits and the potential strategies to change them. Our findings, which bring the individual-based view to the concept of habits, support the relevant role of habituation in weight management and health behavior.

### Limitations and strengths of the study

In order to obtain a new, deeper understanding of lifelong weight management over the life course, we approached the research topic from a qualitative perspective. As qualitative research methods are generally used for understanding views and perceptions and are intended for scarcely studied fields [17], we considered this approach optimal for the purposes of the present study. Another advantage of qualitative research is its ability to identify experiences in people’s everyday lives throughout the life course.

As we wanted to learn about the lifelong weight management of individuals of normal weight, we intentionally selected our participants from among individuals whose experiences and history we expected to yield significant information for our research questions. Naturally, we had no long-term data on weight, and we needed to rely on the interviewees’ reports. However, we excluded two participants who reported during the interview that they had had a weight-gain period. As the study did not aim to generalize the results, we chose an elective sampling method. We believe that in this kind of qualitative study the representativeness of the sample needs to be considered on a basic level, and therefore, we wanted to exclude systematic bias of selection. Consequently, we selected all volunteer candidates who met the recruitment criteria, but the researchers did not deliberately influence the selection process.

As the starting point of the study was a deeper understanding of lifelong successful weight management, we only included normal weight, gender and age in the criteria for participation. However, it is well-known that socioeconomic status is closely associated with health behavior [41]. The majority of our participants (28 for 39 individuals) had studied for over 13 years, and only three belonged to the lowest education category (education of less than 9 years). Socioeconomic background might have modified the results of the study, and the themes describing successful weight management may have been different if the majority of the participants had belonged to the lowest education category. However, our interviewees also disclosed events and routines from childhood that did not support a healthy lifestyle. Moreover, they revealed that not all their families had belonged to the upper-middle class and not all had had a privileged childhood. Thus, an “ideal” lifelong environment did not seem to be necessary for developing a healthy lifestyle.

Further, the tendency to answer in a socially acceptable way may be considered a weakness of our interviews [47]. However, our participants openly discussed the issues in their interviews and did not avoid topics related to, for example, unhealthy eating habits or unhealthy food choices. Another aspect of research reliability is linked to the personal narratives that the interviewees gave on topics related to childhood and transitions. Narrative theory states that interviewees order their memories along coherent narrative structures and plots and tell their narratives in a specific cultural context, which defines a range of possible stories to explain the phenomenon [48]. This is especially typical in narratives describing life transitions. Our participants characterized how they had managed to adjust their lifestyles to altered situations in several ways. It might be that these adaptations were not actually as simple or easy as the interviewees presented. However, it is interesting that our participants’ stories are success stories of life experiences that are typically reported to be the cause of weight gain by most of the Western culture.

The present findings describe the views of participants from the metropolitan area of Finland. Weight management in urban areas might be more favorable than that in rural areas due to frames such as physical and social environments. Rural areas may also have more barriers to women’s weight management, such as a lack of childcare or longer commuting, which deprive weight management resources [6]. However, as our study included both men and women, and individuals from two age groups, it considered weight management from a broader viewpoint.

## Conclusion

The present study offers a novel and important perspective of lifelong weight management. We interpret successful weight management as a journey over the life course. The building of a lifestyle begins in childhood, and continues throughout the life course. These findings encourage us to pay more attention to childhood as a creator of habits and healthy lifestyles. Food-upbringing and an active lifestyle are key issues for preventing obesity: Providing nutrition and exercise education to parents when expecting their first child would be justified, at least for those at a high risk of overweight and obesity.

However, it seems that the experiences of childhood alone are not decisive in terms of success. High self-efficacy in issues related to weight management is likely to play an essential role in maintaining a healthy lifestyle. These findings could inspire obesity treatment programs to concentrate on the factors involved in the development of self-efficacy. Although long-term successful weight management is typically seen as laborious and unattainable by the public, our results demonstrate positive insights into lifelong weight management from an individual’s perspective. Successful weight management did not require continuous work, periods of fasting or extreme exercise; instead it required routines, habits and skills to improve an adopted lifestyle.

## Supplementary information

**Additional file 1.** The interview guide includes the discussion topics that the interviews covered.

**Additional file 2.** The coding book includes information about the categorization of the codes and the main categories. There are also example quotations of every codes.

## Data Availability

The datasets generated and/or analyzed during the current study are not publicly available as they are confidential, but can be obtained from the corresponding author on reasonable request.

## Abbreviation

BMI: Body mass index

## Footnotes

1 Informant 22, Woman, 34 years
