# Supplementary material for: Exploration of Finnish adults’ successful weight management over the life course: a qualitative study
Source: BMC Public Health. 2020 Jan 6;20:12. doi: 10.1186/s12889-019-8128-8 (PMC6945519; doi:10.1186/s12889-019-8128-8)
Supplement: Supplementary file 1 — Additional file 1. The interview guide includes the discussion topics that the interviews covered. [file 12889_2019_8128_MOESM1_ESM.docx]

Additional file 1. Interview guide

Theme interview: Successful weight management

Introduction of research theme, confidence

1. How would you describe your relationship with food?
   1. What is your favorite food? What else do you eat?
   2. Could you give an example of food memory/memories of your life?
   3. What has your relationship with food been like over time?
   4. What does food mean to you?
   5. Which things affect your eating habits?
   6. How do you feel about cooking? Baking?
   7. How often do you eat at a restaurant? For what reason? What does it mean to you?
2. How would you describe your relationship with eating and eating situations?
   1. Why do you eat? When do you stop eating?
   2. How do you pace your meals throughout the day? Please describe this.
   3. Please describe your ordinary day, including meals.
   4. How do parties with buffets affect your eating?
   5. How do holidays and weekends affect your eating?
   6. How do your family/friends affect your eating?
3. What do you think of eating management?
   1. How would you define eating management?
   2. What is important in eating management?
   3. Do you face any eating management challenges?
   4. Do you feel you need to restrict your eating?
   5. Do emotions affect your eating?
   6. What do you think about diets?
   7. How have you succeeded in eating management?
4. What does successful weight management mean to you?
   1. How do you understand weight management?
   2. What do you think is successful weight management?
   3. Do you feel that you need to work on your weight management?
      1. For what reason?
   4. How do you see your weight?
      1. What is your own ideal weight?
      2. How do you track your weight? Do you weigh yourself?
      3. What is your weight history, since childhood?
      4. What do you do if you gain weight?
   5. Could you describe your parents and your siblings’ weight? The weight of your spouse and children?
   6. How have you managed to stay at a normal weight? Which things have affected this?
   7. Could you give an example of successful/unsuccessful weight management?
5. What facilitates weight management? What are the means for weight management?
   1. What do you think of exercise? What is its role in weight management? Why do you exercise?
   2. What role do the people close to you play in weight management?
   3. Name some concrete means for weight management.
   4. Weight management and health behavior (nutrition, physical activity, smoking, use of alcohol)?
   5. Do you face any health behavior challenges?
   6. How do you think weight management affects your life?
   7. Have you experienced adversities in your life?
