# Supplementary material for: Exploration of Finnish adults’ successful weight management over the life course: a qualitative study
Source: BMC Public Health. 2020 Jan 6;20:12. doi: 10.1186/s12889-019-8128-8 (PMC6945519; doi:10.1186/s12889-019-8128-8)
Supplement: Supplementary file 2 — Additional file 2. The coding book includes information about the categorization of the codes and the main categories. There are also example quotations of every codes. [file 12889_2019_8128_MOESM2_ESM.docx]

Additional file 2: Coding book

| **MAIN CATEGORY** | **CODES** | **EXAMPLE QUOTATIONS** |
| --- | --- | --- |
| ADOPTION OF LIFESTYLE |  |  |
|  | CHILDHOOD | “Upbringing is the main element that influences my food habits. I can’t think of any other factors, I’ve noticed that I eat the same foods as I did in childhood. I also reflect on what my mom and dad taught me about food”  “I’ve had an active lifestyle since childhood…I didn’t spend time on the computer or play video games, but exercised frequently and ate regularly and most likely fairly well” |
|  | LEARNING | “I hardly think about weight management anymore …I don’t feel at all that I should restrict my eating or my life or anything, because those patterns are now so internalized…but it didn’t happen by chance…in the beginning, I decided to behave in a way that promotes staying at a normal weight…and now the routines just happen” |
| MAINTENANCE OF LIFESTYLE |  |  |
|  | TRANSITION | “When I was a young man, I ate differently, a lot of unhealthy processed foods, hamburgers and hot-dogs…when I had children, I changed my diet…now it resembles my childhood diet, which included plenty of healthy elements” |
|  | ADULTHOOD | “…I am so routinized, and we have a basic set that we always eat…”  “Well, my husband quite often eats chips on weekends, I typically take two handfuls, and then I quit…I make my decisions…of course, the environment has some influence on people’s patterns, but I think, ultimately, you yourself are in charge of your eating” |
|  | ADJUSTING | “When I began this desk job, I noticed (from my body) that I didn’t need to eat as much as I used to. My previous work had been physically harder, and at that point, I had to consciously “wake myself up” to realize that I would survive with less food…meals didn’t need to be huge…in conclusion, I’ve always found the right balance for my needs in this kind of situation.” |
